# *minet*: A R/Bioconductor Package for Inferring Large Transcriptional Networks Using Mutual Information

**DOI:** 10.1186/1471-2105-9-461

**Published:** 2008-10-29

**Authors:** Patrick E Meyer, Frédéric Lafitte, Gianluca Bontempi

**Affiliations:** 1 Machine Learning Group, Computer Science Department, Faculty of Science, Université Libre de Bruxelles, 1050 Brussels, Belgium

## Abstract

**Results:**

This paper presents the R/Bioconductor package *minet *(version 1.1.6) which provides a set of functions to infer mutual information networks from a dataset. Once fed with a microarray dataset, the package returns a network where nodes denote genes, edges model statistical dependencies between genes and the weight of an edge quantifies the statistical evidence of a specific (e.g transcriptional) gene-to-gene interaction. Four different entropy estimators are made available in the package *minet *(empirical, Miller-Madow, Schurmann-Grassberger and shrink) as well as four different inference methods, namely relevance networks, ARACNE, CLR and MRNET. Also, the package integrates accuracy assessment tools, like F-scores, PR-curves and ROC-curves in order to compare the inferred network with a reference one.

**Conclusion:**

The package *minet *provides a series of tools for inferring transcriptional networks from microarray data. It is freely available from the Comprehensive R Archive Network (CRAN) as well as from the Bioconductor website.

## Background

Modelling transcriptional interactions by large networks of interacting elements and determining how these interactions can be effectively learned from measured expression data are two important issues in system biology [1]. It should be noted that by focusing only on transcript data, the inferred network should not be considered as a proper biochemical regulatory network, but rather as a gene-to-gene network where many physical connections between macromolecules might be hidden by short-cuts. In spite of some evident limitations the bioinformatics community made important advances in this domain over the last few years [2,3]. In particular, mutual information networks have been succesfully applied to transcriptional network inference [4-6]. Such methods, which typically rely on the estimation of mutual information between all pairs of variables, have recently held the attention of the bioinformatics community for the inference of very large networks (up to several thousands nodes) [4,7-9].

R is a widely used open source language and environment for statistical computing and graphics [10] which has become a *de-facto *standard in statistical modeling, data analysis, biostatistics and machine learning [11]. An important feature of the R environment is that it integrates generic data analysis and visualization functionalities with off-the-shelf packages implementing the latest advances in computational statistics. Bioconductor is an open source and open development software project for the analysis and comprehension of genomic data [12] mainly based on the R programming language. This paper introduces the new R and Bioconductor package *minet*, where the acronym stands for *Mutual Information NETwork inference*. This package is freely available on the R CRAN package resource [10] as well as on the Bioconductor website [12].

## 1 Mutual information networks

Mutual information networks are a subcategory of network inference methods. The rationale of this family of methods is to infer a link between a couple of nodes if it has a high score based on mutual information [9].

Mutual informaton network inference proceeds in two steps. The first step is the computation of the mutual information matrix (MIM), a square matrix whose *i, j*-th element

(1) *M I M*_*ij *_= *I*(*X*_*i*_; *X*_*j*_)

is the mutual information between *X*_*i *_and *X*_*j*_, where *X*_*i *_∈ X, *i *= 1,...,*n*, is a discrete random variable denoting the expression level of the *i*th gene. The second step is the computation of an edge score for each pair of nodes by an inference algorithm that takes the MIM matrix as input.

The adoption of mutual information in network inference tasks can be traced back to the Chow and Liu's tree algorithm [13,14]. Mutual information provides a natural generalization of the correlation since it is a non-linear measure of dependency. Hence with mutual information generalized correlation networks (relevance networks [7]) and also conditional independence graphs (e.g. ARACNE [8]) can be built. An advantage of these methods is their ability to deal with up to several thousands of variables also in the presence of a limited number of samples. This is made possible by the fact that the MIM computation requires only $\frac{n(n-1)}{2}$ estimations of a bivariate mutual information term. Since each bivariate estimation can be computed fastly and is low variant also for a small number of samples, this family of methods is adapted for dealing with microarray data. Note that since mutual information is a symmetric measure, it is not possible to derive the direction of an edge using a mutual information network inference technique. Notwithstanding the orientation of the edges can be obtained by using algorithms like IC which are well known in the graphical modelling community [15].

### 1.1 Relevance Network

The relevance network approach [7] has been introduced in gene clustering and was successfully applied to infer relationships between RNA expressions and chemotherapeutic susceptibility [6]. The approach consists in inferring a genetic network where a pair of genes {*X*_*i*_, *X*_*j*_} is linked by an edge if the mutual information *I*(*X*_*i*_; *X*_*j*_) is larger than a given threshold *I*_0_. The complexity of the method is *O*(*n*^2^) since all pairwise interactions are considered.

Note that this method does not eliminate all the indirect interactions between genes. For example, if gene *X*_1 _regulates both gene *X*_2 _and gene *X*_3_, this would cause a high mutual information between the pairs {*X*_1_, *X*_2_}, {*X*_1_, *X*_3_} and {*X*_2_, *X*_3_}. As a consequence, the algorithm will set an edge between *X*_2 _and *X*_3 _although these two genes interact only through gene *X*_1_.

### 1.2 CLR Algorithm

The CLR algorithm [4] is an extension of the relevance network approach. This algorithm computes the mutual information for each pair of genes and derives a score related to the empirical distribution of the MI values. In particular, instead of considering the information *I*(*X*_*i*_; *X*_*j*_) between genes *X*_*i *_and *X*_*j*_, it takes into account the score $z_{ij}=\sqrt{z_{i}^{2}+z_{j}^{2}}$ where

(2) $$z_{i}=\max\left(0,\frac{I(X_{i};X_{j})-\mu_{i}}{\sigma_{i}}\right)$$

and *μ*_*i *_and *σ*_*i *_are respectively the sample mean and standard deviation of the empirical distribution of the values *I*(*X*_*i*_, *X*_*k*_), *k *= 1,...,*n*. The CLR algorithm was successfully applied to decipher the *E. Coli *TRN [4]. CLR has a complexity in *O*(*n*^2^) once the MIM is computed.

### 1.3 ARACNE

The Algorithm for the Reconstruction of Accurate Cellular Networks (ARACNE) [8] is based on the Data Processing Inequality [16]. This inequality states that, if gene *X*_1 _interacts with gene *X*_3 _through gene *X*_2_, then

*I*(*X*_1_; *X*_3_) ≤ min (*I*(*X*_1_; *X*_2_), *I*(*X*_2_; *X*_3_)).

ARACNE starts by assigning to each pair of nodes a weight equal to the mutual information. Then, as in relevance networks, all edges for which *I*(*X*_*i*_; *X*_*j*_) <*I*_0 _are removed, with *I*_0 _a given threshold. Eventually, the weakest edge of each triplet is interpreted as an indirect interaction and is removed if the difference between the two lowest weights is above a threshold *W*_0_. Note that by increasing *I*_0 _the number of inferred edges is decreased while the opposite effect is obtained by increasing *W*_0_.

If the network is a tree and only pairwise interactions are present, the method guarantees the reconstruction of the original network, once it is provided with the exact MIM. ARACNE's complexity is *O*(*n*^3^) since the algorithm considers all triplets of genes. In [8] the method was able to recover components of the TRN in mammalian cells and outperformed Bayesian networks and relevance networks on several inference tasks [8].

### 1.4 MRNET

MRNET [9] infers a network using the maximum relevance/minimum redundancy (MRMR) feature selection method [17,18]. The idea consists in performing a series of supervised MRMR gene selection procedures where each gene in turn plays the role of the target output.

The MRMR method has been introduced in [17,18] together with a best-first search strategy for performing filter selection in supervised learning problems. Consider a supervised learning task where the output is denoted by *Y *and *V *is the set of input variables. The method ranks the set *V *of inputs according to a score that is the difference between the mutual information with the output variable *Y *(maximum relevance) and the average mutual information with all the previously ranked variables (minimum redundancy). The rationale is that direct interactions (i.e. the most informative variables to the target *Y*) should be well ranked whereas indirect interactions (i.e. the ones with redundant information with the direct ones) should be badly ranked by the method. The greedy search starts by selecting the variable *X*_*i *_having the highest mutual information to the target *Y*. The second selected variable *X*_*j *_will be the one with a high information *I*(*X*_*j*_; *Y*) to the target and at the same time a low information *I*(*X*_*j*_; *X*_*i*_) to the previously selected variable. In the following steps, given a set *S *of selected variables, the criterion updates *S *by choosing the variable

(3) $$X_{j}^{MRMR}=\arg\underset{X_{j}\in V\backslash S}{\max}(u_{j}-r_{j})$$

that maximizes the score

(4) *s*_*j *_= *u*_*j *_- *r*_*j*_,

where *u*_*j *_is a relevance term and *r*_*j *_is a redundancy term. More precisely,

*u*_*j *_= *I*(*X*_*j*_; *Y*)

is the mutual information of *X*_*j *_with the target variable *Y*, and

$$r_{j}=\frac{1}{\left|S\right|}\sum_{X_{k}\in S}I(X_{j};X_{k})$$

measures the average redundancy of *X*_*j *_to each already selected variables *X*_*k *_∈ *S*. At each step of the algorithm, the selected variable is expected to allow an efficient trade-off between relevance and redundancy. It has been shown in [19] that the MRMR criterion is an optimal "pairwise" approximation of the conditional mutual information between any two genes *X*_*i *_and *X*_*j *_given the set *S *of selected variables *I*(*X*_*i*_; *X*_*j*_|*S*).

The MRNET approach consists in repeating this selection procedure for each target gene by putting *Y *= *X*_*i *_and *V *= *X *\ {*X*_*i*_}, *i *= 1,...,*n*, where *X *is the set of the expression levels of all genes. For each pair {*X*_*i*_, *X*_*j*_}, MRMR returns two (not necessarily equal) scores *s*_*i *_and *s*_*j *_according to (4). The score of the pair {*X*_*i*_, *X*_*j*_} is then computed by taking the maximum of *s*_*i *_and *s*_*j*_. A specific network can then be inferred by deleting all the edges whose score lies below a given threshold *I*_0 _(as in relevance networks, CLR and ARACNE). Thus, the algorithm infers an edge between *X*_*i *_and *X*_*j *_either when *X*_*i *_is a well-ranked predictor of *X*_*j *_(*s*_*i *_> *I*_0_) or when *X*_*j *_is a well-ranked predictor of *X*_*i *_(*s*_*j *_> *I*_0_).

An effective implementation of the best-first search for quadratic problems is available in [20]. This implementation demands an *O*(*f *× *n*) complexity for selecting *f *features using a best first search strategy. It follows that MRNET has an *O*(*f *× *n*^2^) complexity since the feature selection step is repeated for each of the *n *genes. In other terms, the complexity ranges between *O*(*n*^2^) and *O*(*n*^3^) according to the value of *f*. In practice the selection of features stops once a variable obtains a negative score.

#### Implementation of the inference algorithms in *minet*

All the algorithms discussed above are available in the *minet *package. The RELNET algorithm is implemented by simply running the command build.mim which returns the MIM matrix which can be considered as a weighted adjacency matrix of the network. CLR, ARACNE and MRNET are implemented by the commands aracne(mim), clr(mim), mrnet(mim) respectively that return a weighted adjacency matrix of the network.

It should be noted, that the modularity of the *minet *package makes possible to assess network inference methods on similarity matrices other than MIM [21].

## 2 Mutual information estimation

An information-theoretic network inference technique aims at identifying connections between two genes (variables) by estimating the amount of information common to any pair of genes. Mutual information is a measure which calculates dependencies between two discrete random variables. An important property of this measure is that it is not restricted to the identification of linear relations between the random variables [16].

If *X *is a continuous random variable taking values between *a *and *b*, the interval [*a, b*] can be discretized by partitioning it into |X| subintervals, called *bins*, where the symbol X denotes the bin index vector. We use also *nb*(*x*_*k*_) to denote the number of data points in the *k*th bin and the symbol $m=\sum_{k\in X}nb(x_{k})$ to denote the number of samples. If *X *is a random vector each element *X*_*i *_can be discretized separately into |$X_{i}$| bins with index vector $X_{i}$.

Let *X *be a random vector and *p *a probability measure. The *i, j*-th element of the mutual information matrix (MIM) is defined by

(5) MIMij=H(Xi)+H(Xj)−H(Xi,Xj)=I(Xi;Xj)=∑ki∈Xi∑kj∈Xjp(xki,xkj)log⁡(p(xki,xkj)p(xki)p(xkj)),

where the entropy of a random variable *X *is defined as

(6) $$H(X)=-\sum_{k\in X}p(x_{k})\log p(x_{k})$$

and *I*(*X*_*i*_; *X*_*j*_) is the mutual information between the random variables *X*_*i *_and *X*_*j*_.

Hence, each mutual information calculus demands the estimation of three entropy terms (Eq. 5). A fast entropy estimation is therefore essential for an effective network inference based on MI. Entropy estimation has gained much interest in feature selection and network inference over the last decade [22]. Most approaches focus on reducing the bias inherent to entropy estimation. In this section, some of the fastest and most used entropy estimators are stressed. Other interesting approaches can be found in [22-26].

### 2.1 Empirical and Miller-Madow corrected estimators

The empirical estimator (also called "plug-in", "maximum likelihood" or "naïve", see [23]) is the entropy of the empirical distribution.

(7) $${\hat{H}}^{emp}=-\sum_{k\in X}\frac{nb(x_{k)}}{m}\log\frac{nb(x_{k})}{m}.$$

Note that, because of the convexity of the logarithmic function, an underestimate of *p*(*x*_*k*_) causes an error on *H*(*X *= *x*_*k*_) that is larger than the one given by an overestimation of the same quantity. As a result, entropy estimators are biased downwards, that is

(8) $$E[{\hat{H}}^{emp}(pX)]\leq H(pX).$$

It has been shown that the variance of the empirical estimator is upper-bounded by $var({\hat{H}}^{emp})\leq \left(\frac{{\left(\log m\right)}^{2}}{m}\right)$ which depends only on the number of samples whereas the asymptotic bias of the estimate $bias({\hat{H}}^{emp})=-\frac{|X|-1}{2m}$ depends also on the number of bins |X| [23]. As |X| ≫ *m*, this estimator can still have a low variance but the bias can become very large [23].

The Miller-Madow correction is then given by the following formula which is the empirical entropy corrected by the asymptotic bias,

(9) $${\hat{H}}^{mm}={\hat{H}}^{emp}+\frac{|X|-1}{2m}.$$

where |X| is the number of bins with non-zero probability. This correction, while adding no computational cost to the empirical estimator, reduces the bias without changing variance. As a result, the Miller-Madow estimator is often preferred to the naive empirical entropy estimator.

### 2.2 Shrink entropy estimator

The rationale of the shrink estimator, [27], is to combine two different estimators, one with low variance and one with low bias, by using a weighting factor *λ *∈ [0,1]

(10) $${\hat{p}}_{\lambda }(x_{k})=\lambda \frac{1}{\left|X\right|}+(1-\lambda )\frac{nb(x_{k})}{m}.$$

Shrinkage is a general technique to improve an estimator for a small sample size [3]. As the value of *λ *tends to one, the estimated entropy is moved toward the maximal entropy (uniform probability) whereas when *λ *is zero the estimated entropy tends to the value of the empirical one.

Let λ* be the value minimizing the mean square function, see [27],

(11) $$\lambda *=\arg\underset{\lambda \in [0,1]}{\min}E\left[\sum_{k\in X}\left({\hat{p}}_{\lambda }(x_{k}\right)-p(x_{k}){)}^{2}\right].$$

It has been shown in [28] that the optimal *λ *is given by

(12) $$\lambda *=\frac{\left|X|(m^{2}-\sum_{k\in X}nb{\left(x_{k}\right)}^{2}\right)}{\left(m-1)(|X\text{|}\sum_{k\in X}nb{\left(x_{k}\right)}^{2}-m^{2}\right)}.$$

(13) $${\hat{H}}^{shrink}=-\sum_{k\in X}{\hat{p}}_{\lambda }(x_{k})\log{\hat{p}}_{\lambda }(x_{k)}$$

### 2.3 The Schurmann-Grassberger Estimator

The Dirichlet distribution can be used in order to estimate the entropy of a discrete random variable. The Dirichlet distribution is the multivariate generalization of the beta distribution. It is also the conjugate prior of the multinomial distribution in Bayesian statistics. More precisely, the density of a Dirichlet distribution takes the following form

(14) $$f(X;\beta )=\frac{\prod_{k\in X}\Gamma (\beta_{k})}{\Gamma \left(\sum_{k\in X}\beta_{k}\right)}\prod_{k\in X}x_{k}^{\beta_{k-1}}$$

where *β*_*i *_is the prior probability of an event *x*_*i *_and Γ(·) is the gamma function, (see [25,27,29] for more details).

In case of no a priori knowledge, the *β*_*k *_are assumed to be equal (*β*_*k *_= *N*, *k *∈ X) so as no event becomes more probable than another. Note that using a Dirichlet prior with parameters *N *is equivalent to adding *N *≥ 0 "pseudo-counts" to each bin *i *∈ X. The prior actually provides the estimator the information that |X|*N *counts have been observed in previous experiments. From that viewpoint, |X|*N *becomes the a priori sample size.

The entropy of a Dirichlet distribution can be computed directly with the following equation:

(15) $${\hat{H}}^{dir}(X)=\frac{1}{m+|X|N}\sum_{k\in \text{X}}\left(nb(x_{k})+N)(\psi (m+|X|N+1)-\psi (nb(x_{k})+N+1)\right)$$

with $\psi (z)=\frac{d\ln\Gamma (z)}{dz}$ the digamma function.

Various choices of prior parameters has been proposed in the literature [29-31]. Schurmann and Grassberger have proposed the prior $N=\frac{1}{\left|X\right|}$[32] that has been retained in the package.

#### Implementation of estimators in *minet*

The mutual information matrix is estimated by using the function build.mim(dataset, estimator). This function returns a matrix of paired mutual informations computed in nats (base *e*) and takes two arguments:

1. the data frame dataset which stores the gene expression dataset or a generic dataset where columns contain variables/features and rows contain outcomes/samples

2. the string mi, that denotes the routine used to perform mutual information estimator.

The package makes available four estimation routines : "mi.empirical", "mi.shrink", "mi.sg","mi.mm" (default:"mi.empirical") each referring to the estimators technique explained above.

## 3 Discretization methods

All the estimators discussed in the previous section have been designed for discrete variables. If the random variable *X *is continuous and takes values comprised between *a *and *b*, it is then required to partition the interval [*a, b*] into |X| sub-intervals in order to adopt a discrete entropy estimator. The two most used discretizing algorithm are the equal width and the equal frequency quantization. These are explained in the next sections. Other discretization methods can be found in [33-35].

### 3.1 Equal Width

The principle of the equal width discretization is to divide the range [*a*_*i*_, *b*_*i*_] of each variable *X*_*i*_, *i *∈ {1, 2,...,*n*} in the dataset into |$X_{i}$| sub-intervals of equal size: $[a_{i,}a_{i}+\frac{b_{i}-a_{i}}{\left|X_{i}\right|}[,[a_{i}+\frac{b_{i}-a_{i}}{\left|X_{i}\right|},a_{i}+2\frac{b_{i}-a_{i}}{\left|X_{i}\right|}[,...[a_{i}+\frac{\left(|X_{i}|-1)(b_{i}-a_{i}\right)}{\left|X_{i}\right|},b_{i}+\varepsilon [$. Note that an *ε *is added in the last interval in order to include the maximal value in one of the |$X_{i}$| bins. This discretization scheme has a *O*(*m*) complexity cost (by variable).

### 3.2 Global Equal Width

The principle of the global equal width discretization is the same as the equal width (Sec. 3.1) except that the considered range [*a, b*] is not the range of each random variable such as in Sec. 3.1 but the range of the random vector composed of all the variables in the dataset. In other words, *a *and *b *are respectively the minimal and the maximal value of the dataset.

### 3.3 Equal Frequency

The equal frequency discretization scheme consists in partitioning the range [*a*_*i*_, *b*_*i*_] of each variable *X*_*i *_in the dataset into |$X_{i}$| intervals, each having the same number *m*/|$X_{i}$| of data points points. As a result, the size of each interval can be different. Note that if the |$X_{i}$| intervals have equal frequencies, the computation of entropy is straightforward: it is log $\frac{1}{\left|X_{i}\right|}$. However, there can be more than *m*/|$X_{i}$| identical values in a vector of measurements. In such case, one of the bins will be more dense than the others and the resulting entropy will be different of log $\frac{1}{\left|X_{i}\right|}$. It should be noted that this discretization is reported in some papers as one of the most efficient method (e.g. for naive Bayes classification) [35].

#### Implementation of discretization strategies in *minet*

The discretization is performed in *minet *by the function

discretize(dataset, disc = "equalfreq", nbins = sqrt(nrow(dataset)))

where

• dataset is the dataset to be discretized

• disc is a string which can take three values: "equalfreq" "equalwidth" "globalequalwidth"(default is " equalfreq").

• nbins, the number of bins to be used for discretization, which is by default set to $\sqrt{m}$ with *m *is the number of samples [35]. Note that there are functions used by the built-in R hist() function that can be used here such as nclass. FD(dataset), nclass. scott(dataset) and nclass. Sturges(dataset).

## 4 Assessment of the network inference algorithm

A network inference problem can be seen as a binary decision problem where the inference algorithm plays the role of a classifier: for each pair of nodes, the algorithm either returns an edge or not. Each pair of nodes can thus be assigned a positive label (an edge) or a negative one (no edge).

A positive label (an edge) predicted by the algorithm is considered as a true positive (TP) or as a false positive (FP) depending on the presence or not of the corresponding edge in the underlying true network, respectively. Analogously, a negative label is considered as a true negative (TN) or a false negative (FN) depending on whether the corresponding edge is present or not in the underlying true network, respectively. Note that all mutual information network inference methods use a threshold value in order to delete the arcs having a too low score. Hence, for each treshold value, a confusion matrix can be computed.

### 4.1 ROC curves

The false positive rate is defined as

$$FPR=\frac{FP}{TN+FP},$$

and the true positive rate as

$$TPR=\frac{TP}{TP+FN},$$

also known as recall or sensitivity.

A Receiver Operating Characteristic (ROC) curve, is a graphical plot of the TPR (true positive rate) vs. FPR (false positive rate) for a binary classifier system as the threshold is varied [36]. A perfect classifier would yield a point in the upper left corner (having coordinates [0,1]) of the ROC space, representing 100% TPR (all true positives are found) and 0% FPR (no false positives are found). A completely random guess gives a point along the diagonal line (the so-called line of no-discrimination) which goes from the left bottom to the top right corners. Points above the diagonal line indicate good classification results, while points below the line indicate wrong results.

### 4.2 PR curves

It is generally recommended [37] to use receiver operator characteristic (ROC) curves when evaluating binary decision problems in order to avoid effects related to the chosen threshold. However, ROC curves can present an overly optimistic view of an algorithm's performance if there is a large skew in the class distribution, as typically encountered in transcriptional network inference because of sparseness. To tackle this problem, precision-recall (PR) curves have been cited as an alternative to ROC curves [38].

Let the precision quantity

$$p=\frac{TP}{TP+FP},$$

measure the fraction of real edges among the ones classified as positive and the recall quantity

$$r=\frac{TP}{TP+FN},$$

also know as true positive rate (TPR), denote the fraction of real edges that are correctly inferred. These quantities depend on the threshold chosen to return a binary decision. The PR curve is a diagram which plots the precision (*p*) versus recall (*r*) for different values of the threshold on a two-dimensional coordinate system.

### 4.3 F-Scores

Note that a compact representation of the PR diagram is returned by the maximum and/or the average of the F-score quantity [39]:

$$F=\frac{2pr}{r+p},$$

which is an harmonic average of precision and recall.

The general formula for non-negative real *β *is:

$$F_{\beta }=\frac{\left(1-\beta )(pr\right)}{\beta p+r}$$

where *β *is a parameter denoting the weight of the recall. Two commonly used F-scores are the *F*_2_-measure, which weights recall twice as much as precision, and the *F*_0.5_-measure, which weights precision twice as much as recall. In transcriptional network inference, precision is often a more desirable feature than recall since it is expensive to investigate if a gene regulates another.

#### Assesment functionalities in *minet*

In order to benchmark the inference methods, the package provides a number of assessment tools. The validate(net, ref.net, steps = 50) function allows to compare an inferred network net to a reference network ref.net, described by a Boolean adjacency matrix. The assessment process consists in removing the inferred edges having a score below a given threshold and in computing the related confusion matrix, for steps thresholds ranging from the minimum to the maximum value of edge weigths. A resulting dataframe table containing the list of all the steps confusion matrices is returned and made available for further analysis.

**Figure 3:**
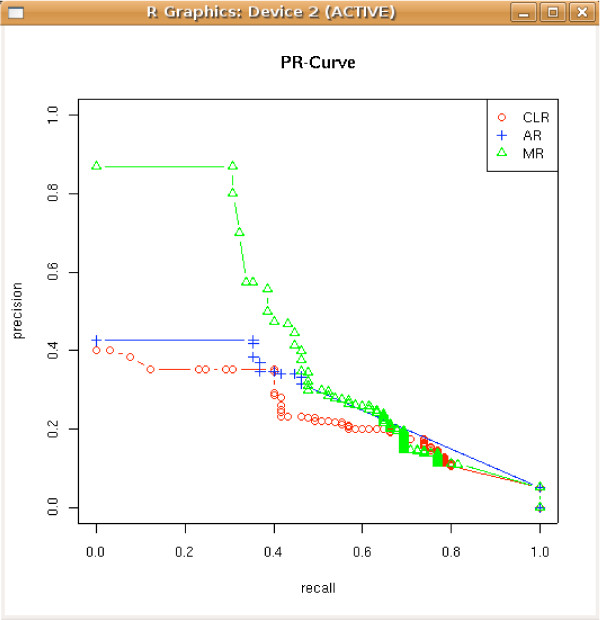
Precision-Recall curves plotted with show.pr(table).

In particular, the function pr(table) returns the related precisions and recalls, rates(table) computes true positive and false positive rates while the function fscores(table, beta) returns the *F*_*β *_– *scores*. The functions show.pr(table) and show.roc(table) allow the user to plot PR-curves and ROC-curves respectively (Figure 3) from a list of confusion matrices.

## 5 Example

Once the R platform is launched, the package, its description and its vignette can be loaded using the following commands:

library(minet)

library(help = minet)

vignette("minet")

A demo script (demo(demo)) shows the main functionalities of the package that we describe in the following.

In order to infer a network with the *minet *package, four steps are required:

• data discretization,

• MIM computation,

• network inference,

• normalization of the network (optional).

**Figure 1:**
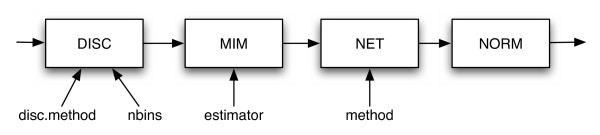
**The four steps in the minet function (discretization disc, mutual information matrix build.mim, inference **mrnet, aracne, clr **and normalization norm.**

The main function of the package is minet which sequentially executes the four steps mentioned above, see Figure 1).

The function minet(dataset, method, estimator, disc, nbins) takes the following arguments: dataset, a matrix or a dataframe containing the microarray data, method, the inference algorithm (such as ARACNE, CLR or MRNET), estimator, the entropy estimator used for the computation of mutual information (empirical, Miller-Madow, shrink, Schurmannn-Grassberger), disc the binning algorithm (i.e. equal frequency or equal size interval) and the parameter nbins which sets the number of bins to use. The final step of the minet function is the normalization using the norm(net) function. This step normalizes all the weights of the inferred adjancy matrix between 0 and 1. Hence, the minet function returns the inferred network as a weighted adjacency matrix with values ranging from 0 to 1 where the higher is a weight, the higher is the evidence that a gene-gene interaction exists.

For demo purposes the package makes available also the dataset syn.data representing the expression of 50 genes in 100 experiments. This dataset has been synthetically generated from the network syn.net using the microarray data generator *Syntren *[40]. This dataset can be loaded with data(syn.data) and the corresponding original network with data(syn.net).

Note that the command res<-minet(syn.data,"mrnet","mi.shrink","equalwidth",10) is a compact way to execute the following sequence of instructions:

discdata<-discretize(syn.data,"equalwidth",10)

mim<-build.mim(discdata,"mi.shrink")

net<-mrnet(mim)

res<-norm(net)

In order to plot a PR-curve (see Figure 3), the functions show.pr and validate can be used.

table <- validate(res, syn.net)

show.pr(table)

**Figure 2:**
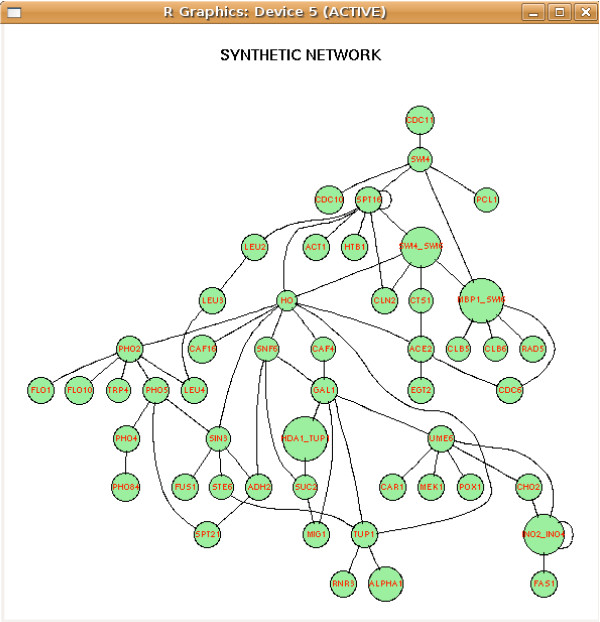
Graph generated with *minet *and plotted with *Rgraphviz*.

In order to display the inferred network, the *Rgraphviz *package [41] can be used with the following commands (see Fig. 2):

library(Rgraphviz)

graph <- as(res, "graphNEL")

plot(graph)

Note that, for the sake of computational efficiency, all the inference functions as well as the entropy estimators are implemented in C++. As a reference, a network of five hundreds variables may be inferred in less than one minute on an Intel Pentium 4 with 2 Ghz and 512 DDR SDRAM.

## 6 Conclusion

Transcriptional network inference is a key issue toward the understanding of the relationships between the genes of an organism. Notwithstanding, few public domain tools are available once a thourough comparison of existing approaches is at stake. A new R/Bioconductor package, freely available, has been introduced in this paper. This package makes available to biologists and bioinformatics practicioneers a set of tools to infer networks from microarray datasets with a large number (several thousands) of genes. Four information-theoretic methods of network inference (i.e. Relevance Networks, CLR, ARACNE and MRNET), four different entropy estimators (i.e. empirical, Miller-Madow, Schurmann-Grassberger and shrink) and three validation tools (i.e. F-scores, PR curves and ROC curves) are implemented in the package. We deem that this tool is an effective answer to the increasing need of comparative tools in the growing domain of transcriptional network inference from expression data.

## Authors' contributions

PEM and FL carried out the implementation of the R package *minet *(up to version 1.1.6). PEM and GB have written the package documentation as well as the manuscript. All authors read and approved the final version of the manuscripts.

## Availability and requirements

The R-package *minet *is freely available from the Comprehensive R Archive Network (CRAN) at  as well as from the Bioconductor website . The package runs on Linux, Mac OS and MS Windows using an installed version of R.

**Table 1:** Available functions of the package *minet *(version 1.1.6)

Function	Usage
minet(data, method, estimator, disc, nbins)	Network inference from data

discretize(data, disc, nbins)	Unsupervised discretization

build.mim(data, estimator)	Mutual information matrix estimation Estimator can be ""mi.empirical","mi.mm","mi.shrink" and "mi.sg".

mrnet(mim)	MRNET algorithm

aracne(mim)	ARACNE algorithm

clr(mim)	CLR algorithm

norm(net)	matrix/network normalization

validate(net1, net2, steps)	Computes confusion matrices

pr(table)	Computes precisions and recalls from confusion matrices

rates(table)	Computes true positive rates and false positive rates from confusion matrices

show.pr(table)	Displays precision-recall curves from confusion matrices

show.roc(table)	Displays receiver operator caracteristic curves from confusion matrices

fscores(table)	Returns a vector of *F*_*β*_-scores from confusion matrices
